# Draft Genome Sequence of the Pathogenic Filamentous Fungus *Aspergillus udagawae* Strain IFM 46973^T^

**DOI:** 10.1128/genomeA.00834-15

**Published:** 2015-08-06

**Authors:** Yoko Kusuya, Azusa Takahashi-Nakaguchi, Hiroki Takahashi, Takashi Yaguchi

**Affiliations:** Medical Mycology Research Center, Chiba University, Chiba, Japan

## Abstract

The incidence of aspergillosis by *Aspergillus* infection has dramatically increased in recent years. *Aspergillus udagawae*, a species related to *Aspergillus fumigatus*, is known as an emerging pathogen of aspergillosis. Here, we present the draft genome sequence of *A. udagawae* strain IFM 46973^T^.

## GENOME ANNOUNCEMENT

Aspergillosis is most commonly caused by *Aspergillus* species. The heterothallic ascomycete *Aspergillus udagawae* (former name *Neosartorya udagawae*) was first isolated from the soil of a Brazilian sugar cane plantation (1) and then was reported as a cause of invasive aspergillosis in humans and cats (2, 3) and bronchial aspergillosis (4). *A. udagawae* can grow even at 10°C and optimally at temperatures in the range of 30 to 35°C but fails to grow at >42°C (5). Although *A. udagawae* shows morphological similarity to *A. fumigatus*, which is a major pathogen of aspergillosis among *Aspergillus* species, it has been reported that four *A. udagawae* strains exhibited decreased susceptibility to antifungal agents, i.e., amphotericin B, itraconazole, and voriconazole (2, 6). The molecular mechanisms underpinning the decreased susceptibility remain unknown. The whole-genome sequence of *A. udagawae* may be of help for elucidating the mechanisms resistant to antifungal agents, developing appropriate therapy in aspergillosis, and addressing the genetic diversity of *Aspergillus* species.

The fungal strain used in this study, *A. udagawae* IFM 46973^T^, was stored and maintained at the Medical Mycology Research Center, Chiba University (IFM strains) in Japan. *A. udagawae* genomic DNA extracted from 2-day-old culture by phenol-chloroform extraction and NucleoBond AXG column (TaKaRa) with NucleoBond buffer set III (TaKaRa) was used to generate paired-end and mate-paired libraries. A paired-end library with insert sizes of 700 bp was generated using an S2 sonicator (Covaris) and QIAquick gel extraction kit (Qiagen), followed by NEBNext Ultra DNA library prep kit (New England BioLabs) and NEBNext multiplex oligos (New England BioLabs), according to the manufacturer’s instructions. Mate-paired libraries with insert sizes of 3.5 to 4.5 kb, 5 to 7 kb, and 8 to 11 kb were generated by a gel selection protocol of the Nextera mate pair kit (Illumina) using a 0.6% agarose gel, according to the manufacturer’s instructions. One hundred-base pair paired-end sequencing was performed with the aid of HiSeq 1500 (Illumina) using the HiSeq reagent kit version 1, according to the manufacturer’s instructions.

After removing the adapter sequences, trimming poor-quality bases (Phred score, <25), and eliminating reads <50 bp using fastq-mcf in ea-utils (version 1.1.2-806) (7), a total of 16,261,526 mate-paired and 15,444,665 paired-end reads were retained. A draft of the *A. udagawae* genome sequence was assembled using the software Platanus version 1.2.1 (8). Eventually, 72 scaffolds (>1,000 bp) consisting of 1,029 contigs (>0 bp) were obtained, totaling 32,004,942 bp, with an overall G+C content of 49.68%. The *N*_50_ was 2,909,130 bp, and the maximum scaffold was 4,322,057 bp long. Gene annotation using the AUGUSTUS program (9), trained with the parameters of the species *A. fumigatus*, resulted in 9,999 genes. Additionally, we predicted 180 tRNAs and 35 rRNAs by tRNAscan-SE 1.3.1 (10) and RNAmmer 1.2 (11), respectively.

### Nucleotide sequence accession numbers.

This whole-genome shotgun project has been deposited at DDBJ/EMBL/GenBank under the accession numbers BBXM01000001 to BBXM01001029. The versions described in this paper are the first versions.
